# Recombinant Human TSH Versus Thyroid Hormone Withdrawal: The Role in the Preparation for RAI Therapy in Differentiated Thyroid Cancer: A Comprehensive Evidence-Based Review

**DOI:** 10.3390/jcm14145000

**Published:** 2025-07-15

**Authors:** Motaz Daraghma, Michael M. Graham

**Affiliations:** 1 Department of Radiology, The University of Iowa, Iowa City, IA 52242, USA; michael-graham@uiowa.edu; 2 University of Missouri-Kansas City, Kansas City, MO 64110, USA

**Keywords:** differentiated thyroid cancer, radioactive iodine therapy, thyroid hormone withdrawal, recombinant human TSH

## Abstract

Radioactive iodine (RAI) therapy plays a fundamental role in the management of differentiated thyroid cancer (DTC) following appropriate surgical intervention. High levels of TSH are required in order to achieve maximum RAI uptake in residual thyroid tissue or metastatic cells. The two techniques that are most commonly used are thyroid hormone withdrawal (THW), which induces endogenous TSH elevation by creating a hypothyroid state, and exogenous stimulation with recombinant human TSH (rhTSH). This review compares both approaches over a range of DTC risk categories. Extensive evidence demonstrates that rhTSH and THW yield equivalent oncological outcomes, including remnant ablation success, recurrence-free survival, and overall survival, in low-, intermediate-, and high-risk disease. Additionally, rhTSH maintains quality of life by avoiding hypothyroid symptoms. While THW continues to be an excellent option when there is a lack of availability of rhTSH, its disadvantages, particularly the transient hypothyroid state, must be carefully weighed against the demonstrated equivalence in efficacy. In current clinical practice, rhTSH is frequently the preferred option for its convenience, safety, and patient-centered benefits; however, the selection of the optimal approach should be based on individual clinical circumstances and patients’ preferences, as well as resource considerations.

## 1. Introduction

Differentiated thyroid cancer (DTC) is typically managed with surgery followed by radioactive iodine-131 (RAI) therapy in appropriate risk groups [1]. Effective RAI treatment requires elevated thyroid-stimulating hormone (TSH) levels to maximize the uptake of iodine by residual thyroid tissue or thyroid cancer cells [2]. Elevated TSH upregulates the sodium–iodide symporter (NIS) on thyroid cells, thereby increasing RAI absorption in target lesions [2]. There are two principal methods to achieve the high TSH levels needed for RAI therapy: endogenous TSH stimulation via thyroid hormone withdrawal (THW) and exogenous TSH stimulation using recombinant human TSH (rhTSH, e.g., thyrotropin alfa, brand name Thyrogen) [1,2]. This review provides a comprehensive comparison of these methods, examining their mechanisms, benefits, and drawbacks, and provides guidance on selecting an approach based on clinical context. The relevant literature was identified through a targeted search of PubMed and Google Scholar (2000–2024) using combinations of terms such as “recombinant TSH,” “thyroid hormone withdrawal,” “RAI therapy,” and “differentiated thyroid cancer.” Preference was given to randomized controlled trials, meta-analyses, large retrospective studies, and major clinical guidelines (e.g., from the American Thyroid Association). Additional references were selected based on their clinical relevance to various DTC risk categories, quality-of-life outcomes, safety, and real-world applicability.

## 2. Mechanisms of TSH Elevation

### 2.1. Endogenous Stimulation via Thyroid Hormone Withdrawal

Thyroid hormone withdrawal (THW) is a well-established method for inducing endogenous TSH elevation in patients with DTC following thyroidectomy [3]. After total or near-total thyroidectomy for DTC, patients are normally placed on levothyroxine to suppress TSH. THW involves stopping the exogenous thyroid hormone for several weeks to induce hypothyroidism [3]. As circulating T4/T3 levels fall, the pituitary gland increases TSH secretion (negative feedback effect), typically achieving TSH > 30 mIU/L after 3–4 weeks off levothyroxine [3]. Some protocols use liothyronine (T3) for the first 2 weeks initially, then withdraw T3 for an additional 2 weeks to shorten the hypothyroid period [3]. The result is an endogenous TSH surge that can persist for many days. This endogenous TSH binds TSH receptors on any remaining normal thyroid cells or DTC cells, stimulating NIS expression and iodine uptake [2]. Patients must remain in a hypothyroid state until RAI is administered and is taken up by the target tissue.

### 2.2. Exogenous Stimulation Using Recombinant Human TSH

Recombinant human TSH (rhTSH) offers an alternative strategy by achieving TSH elevation exogenously, without the need for hormone withdrawal and the resulting hypothyroid state [3]. rhTSH is a synthetic form of TSH that is injected intramuscularly to raise TSH without withdrawing the thyroid hormone. The typical regimen is two doses of 0.9 mg of rhTSH given on consecutive days, with RAI therapy administered 24 h after the second dose [4,5,6]. rhTSH produces a rapid spike in serum TSH (often well above 30 mIU/L, comparable to or higher than levels achieved by THW) [3,6]. Exogenous TSH stimulation activates thyroid cell receptors similarly to endogenous TSH, enhancing NIS expression and RAI uptake in thyroid remnants or cancer cells [2]. Because the patient remains on levothyroxine during this process, they avoid hypothyroidism. Notably, rhTSH is effective even in patients who cannot mount an adequate TSH response (for example, those with large-volume residual disease that produces T4 or hypopituitarism) [3]. In summary, THW leverages the body’s own TSH rise due to hormone deprivation, whereas rhTSH directly supplies TSH, achieving the same goal of stimulating iodine uptake in DTC cells by different means.

## 3. Comparative Analysis of Preparation Methods

### 3.1. Impact on Patient Quality of Life

The major difference between THW and rhTSH lies in short-term patient quality of life (QoL). THW induces iatrogenic hypothyroidism, which commonly causes fatigue, lethargy, weight gain, cold intolerance, constipation, depressed mood, and cognitive slowing [1]. Multiple studies have documented that patients undergoing THW experience a significant transient decline in health-related QoL during the hypothyroid period [3]. For example, in five randomized trials assessing QoL around the time of remnant ablation, hypothyroid patients had markedly worse QoL scores and more hypothyroid symptoms than those prepared with rhTSH [7,8,9,10,11]. Short-term hypothyroidism has also been associated with increased depression and anxiety in DTC patients [2].

In contrast, rhTSH allows patients to remain euthyroid, thus avoiding the constellation of hypothyroid symptoms [1]. Patients prepared with rhTSH report significantly better physical and cognitive functioning in the peri-therapy period [1,3]. For instance, a 2024 randomized trial noted a substantially lower incidence of hypothyroid symptoms (weight gain, constipation, etc.) in the rhTSH group compared to the THW group [1]. Several weeks after RAI (once the thyroid hormone is reintroduced for THW patients), longer-term QoL equalizes between the groups [3]. However, the short-term QoL advantage of rhTSH is well-established, leading to improved patient comfort and the ability to continue normal daily activities during RAI preparation [3]. In practical terms, rhTSH-prepared patients can often continue working and have less impairment, whereas THW often causes significant fatigue and cognitive slowing that can interfere with daily life [2]. Thus, patient quality of life is a clear benefit of rhTSH stimulation, sparing patients the morbidity of hypothyroidism.

### 3.2. Treatment Efficacy and Oncologic Outcomes

An essential consideration is if the method of TSH stimulation affects the efficacy of RAI treatment, including successful thyroid remnant ablation, as well as long-term tumor control, recurrence rates, and survival. Extensive evidence over the past two decades indicates that rhTSH and THW provide equivalent treatment efficacy in DTC [3].

#### 3.2.1. Remnant Ablation Success

Multiple randomized controlled trials (RCTs) in postoperative DTC patients (generally low-to-intermediate risk, without distant metastases) have demonstrated noninferiority of rhTSH compared to THW for achieving successful thyroid remnant ablation [3]. A large 2019 French multicenter cohort (ThyrNod study) of 404 patients with nodal metastases (pT1–T3, N1, M0) compared rhTSH (*n* = 205) with THW (*n* = 199) for RAI preparation [12]. At 6–18 months post-treatment, disease-free status was achieved in 75.1% of rhTSH patients vs. 71.9% of THW patients, a difference of 3.3% (95% CI: −6.6 to 13.0), meeting noninferiority criteria (<15% margin) [12]. At the final evaluation (mean follow-up of ~30–37 months), complete response rates remained equivalent: 83.5% for rhTSH and 81.5% for THW [4]. Subgroup analyses found no prognostic factors (age, tumor stage, nodal burden) that influenced outcomes by preparation method [12].

Additionally, a pooled meta-analysis of seven randomized trials involving 1535 patients found no significant difference in ablation success between rhTSH and THW: pooled risk ratio = 0.97 (95% CI: 0.94–1.01, *p* = 0.10), slightly favoring rhTSH [13]. For example, in six RCTs of patients without metastases (tumor stages T1–T3, N0/N1), the rates of successful ablation were statistically equivalent whether patients were prepared with rhTSH or by THW, across RAI doses ranging from 30 to 100 mCi [7,8,9,10,11,14]. Long-term follow-up from these trials has shown no difference in outcomes, including recurrence-free survival.

A recent retrospective study from Taiwan involving 647 DTC patients reported equivalent ablation outcomes between rhTSH and THW. An “excellent response” (defined as negative imaging and suppressed Tg < 0.2 ng/mL) was achieved in 80.5% of THW patients versus 76.5% of rhTSH patients (*p* = 0.221) [15]. Subgroup analyses by age, sex, extrathyroidal extension, lymph node metastasis, and TNM stage showed no statistically significant differences between groups [15]. For example, among high-risk patients (stage III–IV), excellent response rates were 81.8% (THW) vs. 69.8% (rhTSH) (*p* = 0.074). Logistic regression confirmed that extrathyroidal extension, LN metastasis, and I-131 dose—not the TSH stimulation method—were significant predictors of response [15].

These findings reinforce that ablation efficacy is comparable with either method of TSH elevation.

#### 3.2.2. Adjuvant Therapy for Intermediate/High-Risk Disease

For patients with more advanced DTC (beyond low risk), earlier studies were limited, but emerging data in the last five years support comparable efficacy of rhTSH in this setting as well. The 2015 ATA guidelines noted insufficient evidence at that time to definitively recommend rhTSH for RAI treatment in intermediate-risk patients [1,3]. However, in 2024, a large multicenter RCT (TRESON-01 trial) in intermediate-risk DTC demonstrated that rhTSH (using a novel formulation, SNA001) was noninferior to THW in achieving successful RAI adjuvant therapy [1]. In this trial of 307 patients (mostly with T3 and/or N1 disease), RAI success rates were 43.8% with rhTSH vs. 47.1% with THW, meeting the prespecified noninferiority margin [1]. There was no compromise in efficacy with rhTSH even in patients with lymph node metastases (N1 subgroup success: 42.6% rhTSH vs. 45.3% THW) [1]. Additionally, rhTSH patients experienced significantly fewer adverse events during therapy (30% vs. 59%), with notably less weight gain (12% vs. 43%) and constipation (3% vs. 11%) [1].

For higher-risk DTC and those with distant metastases, rhTSH historically was not FDA-approved (due to a lack of early trial data) [16], leading some clinicians to prefer THW in those situations. However, accumulating evidence indicates that outcomes remain equivalent. A 2023 systematic review and meta-analysis of 10 studies, including 1929 patients with metastatic DTC (953 rhTSH and 976 THW), concluded that preparation with rhTSH had no significant impact on I-131 therapy effectiveness compared to THW [2]. The pooled risk ratio for initial response was 1.02 (95% CI: 0.93–1.12, *p* = 0.68), and for disease progression, RR = 0.97 (95% CI: 0.92–1.03, *p* = 0.39), indicating no statistically significant difference between stimulation methods [2]. Also, a 2022 retrospective study of 55 patients with metastatic DTC found no statistically significant difference in progression-free survival (PFS, *p* = 0.13) or overall survival (OS, *p* = 0.85) of patients prepared with rhTSH (*n* = 27) vs. THW (*n* = 28) [16]. Multivariate analysis showed that only older age at diagnosis (HR = 1.047 per year, *p* = 0.003) and total cumulative RAI activity (HR = 1.007 per mCi, *p* = 0.03) were associated with worse survival outcomes [16]. The method of TSH stimulation was not independently associated with either PFS or OS [16]. Additionally, a recent retrospective analysis by Higuchi et al. evaluated 81 intermediate- to high-risk DTC patients with progressive disease [17]. Patients were divided into three groups based on RAI preparation: rhTSH only (*n* = 21), THW only (*n* = 49), and mixed (*n* = 11). After a median follow-up of 83 months, there were no statistically significant differences in final disease status, OS (*p* = 0.06), or PFS (*p* = 0.10) across the groups. The median PFS was 21 months (rhTSH) vs. 38 months (THW), and the median OS was 50 months (rhTSH) vs. 68 months (THW) [17]. Although these differences were not statistically significant, the magnitude may indicate a potential clinical relevance that warrants further investigation. These findings support the noninferiority of rhTSH, even in more aggressive DTC settings.

Overall, both approaches appear to achieve comparable delivery of radiation to tumor tissue. Any theoretical concerns that rhTSH might lead to lower RAI uptake in tumors (due to its shorter TSH elevation) have not translated into worse clinical outcomes [2]. Consequently, treatment efficacy (remnant ablation success, tumor control, and survival) is equivalent between rhTSH and THW in DTC across risk levels, as supported by multiple RCTs, meta-analyses, and long-term cohort data [2].

#### 3.2.3. Disease Recurrence and Survival

Long-term cancer outcomes appear equally good with either TSH stimulation method. A 2024 meta-analysis pooled 10 studies (6 RCTs and 4 observational; *N* = 2833) with at least 2 years of follow-up and found no significant difference in medium- or long-term recurrence rates between rhTSH and THW preparation [18]. Overall recurrence occurred in 9.5% of rhTSH patients vs. 11.0% of THW patients (RR = 1.07, 95% CI: 0.87–1.32; *p* = 0.53) [18]. Subgroup analyses confirmed no difference in recurrence rates among RCTs only (RR = 1.28, *p* = 0.17), observational studies (RR = 0.94, *p* = 0.62), or studies with ≥5 years follow-up (RR = 1.14, *p* = 0.46) [18]. Even structural incomplete response (SIR) rates were not significantly different when limited to RCTs (RR = 1.64, 95% CI: 0.98–2.73; *p* = 0.06), supporting the equivalence of both methods in long-term effectiveness [18]. These findings are supported by a *Lancet* follow-up of low-risk patients and other cohorts, which showed similar recurrence rates whether ablation was performed after rhTSH or after THW (no discernible impact on long-term disease-free survival) [19,20]. Survival outcomes likewise appear comparable. A 2022 retrospective study of 88 patients with distant metastases from papillary thyroid cancer reported 10-year disease-specific survival (DSS) rates of 62.2% (THW) vs. 73.3% (rhTSH)—a difference that was not statistically significant (*p* > 0.05) [21]. Similarly, PFS at 10 years was 38.3% (THW) vs. 43.5% (rhTSH) [12]. Multivariate analysis confirmed that age ≥55 years (*p* = 0.003), male sex (*p* = 0.017), and non-pulmonary metastases (*p* = 0.047) were independently associated with poorer DSS, whereas the method of TSH stimulation was not. Notably, rhTSH preparation was associated with better PFS in RAI-avid patients (10-year PFS: 64.8% with rhTSH vs. 50.9% with THW) [21].

In summary, both methods achieve equivalent RAI efficacy in eliminating residual disease and preventing recurrence across risk levels, including intermediate-risk cases with lymph node metastases [12].

#### 3.2.4. Role of Tumor Histology

Regarding tumor histology, both papillary and follicular thyroid carcinomas (which together comprise the bulk of DTC) respond well to RAI after either preparation—major trials enrolled both types without noting any difference in ablation efficacy by histological subtype [15,22]. Limited data suggest that Hürthle cell carcinoma (a follicular variant that is often less RAI-avid) also shows no clear benefit of one TSH stimulation method over the other, aside from ensuring adequate dose and dosimetry. Medullary and anaplastic thyroid cancers do not take up RAI and thus do not apply to this comparison [15].

Overall, for differentiated histologies that do take up RAI, current evidence and expert consensus indicate no significant efficacy advantage of THW over rhTSH (or vice versa) in most clinical circumstances [2,23].

### 3.3. Radiation Exposure to Non-Target Tissues

#### 3.3.1. Impact of Hypothyroidism on RAI Pharmacokinetics

Radiation exposure to non-target tissues differs notably between THW and rhTSH, primarily due to changes in RAI pharmacokinetics. When a patient is hypothyroid (as in THW), there are physiological changes that can affect RAI pharmacokinetics. Hypothyroidism is known to reduce the glomerular filtration rate (GFR) and renal plasma flow, which slows the clearance of radioiodine from the blood [14]. In contrast, euthyroid patients (as with rhTSH stimulation) have normal renal function during RAI therapy. As a result, there is a faster elimination of RAI in rhTSH-prepared patients and a shorter effective half-life of radioiodine in the body [24].

#### 3.3.2. Differences in Whole-Body Retention and Bone Marrow Dose

Dosimetric studies have consistently shown that whole-body radiation retention is lower with rhTSH compared to THW [24]. For example, one study reported that the average whole-body effective half-life of I-131 was significantly shorter in rhTSH patients (~16 h) versus hypothyroid patients (~22 h) [24]. Many other investigations (using whole-body counting and blood dosimetry) likewise found that patients undergoing THW have longer residence times of RAI and higher total-body radiation doses than those receiving rhTSH [24]. The bone marrow is especially sensitive to circulating radiation, and the longer the RAI circulates, the greater the marrow dose. By accelerating iodine clearance, rhTSH tends to reduce bone marrow radiation exposure for a given administered activity [1]. In fact, using rhTSH, even high therapeutic RAI doses (e.g., 200–300 mCi) can often be given while keeping the bone marrow absorbed dose under recommended safety thresholds (≤2 Gy) [25].

#### 3.3.3. Renal Radiation Burden and Preservation of Kidney Function

The renal system is another critical site of non-target radiation exposure, with distinct effects depending on thyroid status during RAI therapy. The kidneys are the primary route of RAI excretion (via urine) and thus receive radiation as well. In hypothyroid patients, a reduced GFR means the kidneys and whole body are exposed to RAI for a longer period [24]. Patients with already impaired renal function are particularly affected. A dosimetric analysis stratifying DTC patients by GFR found that hypothyroid patients with a GFR < 60 mL/min had a significantly longer whole-body RAI half-life and a higher residual body radiation dose than their rhTSH counterparts [24]. In other words, THW dramatically exacerbated radiation exposure in the setting of renal insufficiency, whereas rhTSH patients had lower exposure even with a low GFR [24]. Importantly, hypothyroid patients in that study experienced an acute drop in the GFR (from ~94 to 76 mL/min on average), whereas those given rhTSH had no change in the GFR (remaining ~91 mL/min) [24]. This illustrates that rhTSH preserves renal function and promotes faster clearance relative to THW.

#### 3.3.4. Salivary Gland Radiation and Potential Protection by rhTSH

The salivary glands, often affected by RAI therapy, may also benefit from rhTSH preparation due to its influence on whole-body clearance. Other non-target organs also benefit from the shorter systemic circulation of RAI with rhTSH. For instance, salivary glands can be damaged by RAI (causing xerostomia and sialadenitis) [26]. Recent research suggests that rhTSH preparation may modestly reduce salivary gland radiation damage compared to THW [26]. One study reported a small but significant long-term preservation of salivary function in patients treated with rhTSH, likely due to the decreased whole-body retention time of radioiodine [26].

#### 3.3.5. Overall Implications for Clinical Practice

Taken together, the dosimetric and clinical data support a safety advantage of rhTSH in minimizing radiation to non-target tissues. Overall, the use of rhTSH tends to lower total-body and bone marrow dose for any given RAI activity [24]. By avoiding hypothyroidism, rhTSH reduces unnecessary radiation to non-target tissues, potentially minimizing side effects like marrow suppression and radiation-induced tissue damage [1]. In contrast, THW leads to more prolonged RAI residence and thus higher off-target radiation exposure. This safety advantage of rhTSH is one reason guidelines favor rhTSH when feasible [1]. In summary, rhTSH stimulation reduces radiation exposure to non-target tissues (bone marrow, kidneys, etc.) through faster clearance, whereas THW can increase exposure due to slowed metabolism and clearance [24].

### 3.4. Time to Treatment and Practical Considerations

#### 3.4.1. Treatment Timeline and Preparation Speed

The logistics and timing of RAI treatment differ meaningfully between THW and rhTSH, which can influence clinical decision-making and patient convenience. Thyroid hormone withdrawal requires a substantial lead time to ensure adequate TSH elevation. Typically, patients must be off levothyroxine for at least 4 weeks (or off T3 for ~2 weeks if using the T3 bridge) before RAI administration [3]. TSH is checked to confirm it has risen above a target threshold (usually >30 mIU/L) prior to giving the radioactive iodine [3]. This means that, from the time of thyroidectomy, one might wait 4–6 weeks to perform RAI ablation using THW (allowing time for wound healing and hormone withdrawal). If RAI is needed for recurrent disease later, a withdrawal period is needed again, delaying therapy.

In contrast, rhTSH allows much faster preparation. RhTSH is typically given over 2 days, and RAI can be administered on day 3. Patients can remain on the thyroid hormone right up until the rhTSH injections, and they can continue the hormone the day after RAI administration [3]. Thus, the overall timeline from decision to treatment with RAI to actual treatment is shorter with rhTSH [3]. For example, if immediate RAI therapy is indicated (such as in a patient with progressive metastatic disease), using rhTSH means one can proceed with RAI within days, rather than waiting weeks for a THW-induced TSH rise. This shorter time to treatment could be critical in rapidly advancing disease [3]. Moreover, rhTSH spares the patient from the prolonged period of hypothyroid symptoms during withdrawal, which can otherwise complicate or delay scheduling (patients on THW might feel too unwell, potentially necessitating support or hospitalization in extreme cases) [3].

#### 3.4.2. Practical Convenience for Patients and Providers

Beyond timing, the overall convenience of RAI preparation also differs meaningfully between the two methods. In terms of convenience, rhTSH is clearly more convenient for patients and providers. Patients still need to endure dietary iodine restriction for the same amount of time (both methods still require a low-iodine diet for about 2 weeks pre-RAI, but only THW adds the burden of weeks of hypothyroidism). With rhTSH, there are two clinic visits for injections, but otherwise, patients can maintain their normal routine. In contrast, THW often entails multiple visits for monitoring and managing hypothyroid symptoms, and patients may require time off work due to fatigue or cognitive effects [2,3,11,23,27].

From the provider perspective, coordinating a withdrawal can be challenging, especially if patients inadvertently take the thyroid hormone or if endogenous TSH does not rise as expected (for instance, if residual thyroid tissue is producing sufficient hormones). rhTSH provides a reliable, predictable TSH elevation without these uncertainties [3]. The only notable inconvenience of rhTSH might be cost and access—rhTSH (Thyrogen) is expensive and may not be covered in all healthcare systems or available in all regions. In contrast, inducing hypothyroidism is “free” from a drug cost standpoint, which historically influenced practice in resource-limited settings. However, many studies have argued that the improved QoL and reduced productivity loss with rhTSH offset its drug cost in cost-effectiveness analyses, especially for low-risk patients [3]. Overall, when available, rhTSH offers a more convenient and timely route to RAI therapy, whereas THW requires a longer, less comfortable preparation period for the patient [3].

Table 1 provides a concise overview of key studies comparing THW and rhTSH in DTC, focusing on their findings for ablation success, clinical outcomes, QoL measures, and safety.

### 3.5. Summary of Key Differences Between rhTSH and THW

To synthesize the evidence discussed in the preceding sections, Table 2 provides a comparative overview of the two primary TSH stimulation methods—rhTSH and thyroid hormone withdrawal—across key clinical domains including efficacy, safety, quality of life, and practical logistics. This summary may assist clinicians in selecting the most appropriate approach based on patient characteristics and care priorities.

## 4. Conclusions

Preparation for RAI therapy in differentiated thyroid cancer can be achieved via endogenous TSH elevation (THW) or rhTSH injections, each effectively increasing TSH to stimulate radioiodine uptake. Thyroid hormone withdrawal has the advantages of not requiring special medication and decades of clinical experience, but it induces a transient hypothyroid state that substantially worsens short-term quality of life and can pose health risks in vulnerable patients.

RhTSH injection, by contrast, allows patients to remain euthyroid, conferring better patient comfort, faster treatment timelines, and reduced radiation exposure to the whole body. Critically, extensive evidence shows no difference in treatment efficacy between the two methods—rhTSH and THW achieve comparable remnant ablation rates, PFS, and OS in DTC.

The choice of method should therefore be guided by patient-centered considerations and clinical context. In a patient with excellent performance status and no contraindications, either method will treat the cancer equally well. However, the aggregate benefits of rhTSH—improved short-term well-being, safety in patients with comorbidities, and convenience—often make it the preferred approach in modern practice.

Thyroid hormone withdrawal remains an important tool, especially where rhTSH is not available or in certain unique situations, but its limitations are significant and should be weighed against the demonstrated equivalence in outcomes. Guidelines increasingly reflect this balance, recommending rhTSH in most scenarios to avoid unnecessary hypothyroidism.

In conclusion, both exogenous and endogenous TSH stimulation are effective means to prepare DTC patients for RAI therapy. The optimal method should be individualized, but for the majority, rhTSH offers a more patient-friendly pathway to successful treatment with no compromise in cancer control. By understanding the mechanisms, evidence, and clinical nuances detailed in this review, clinicians can make informed decisions to maximize the therapeutic benefit while minimizing patient burden in the management of DTC.

## Figures and Tables

**Table 1:** Representative key studies comparing rhTSH and THW for RAI in DTC. Included studies comprise randomized controlled trials (RCTs), meta-analyses, and large retrospective cohorts. This table highlights comparative outcomes across DTC risk categories.

Study Design	Population	Key Findings: rhTSH vs. THW	Reference
RCT	63 DTC patients without distant metastases (T2–T4/N0–N1, M0); all received 100 mCi RAI	Ablation success (no visible uptake or <0.1%) in 100% of both groups. Tg < 2 ng/mL: 96% (rhTSH) vs. 86% (THW); *p* = 0.23. QoL significantly better with rhTSH (Billewicz score: 27 ± 7 vs. 18 ± 4; *p* < 0.0001). Blood radiation dose 35% lower with rhTSH (0.109 vs. 0.167 mGy/MBq; *p* < 0.0001). Supports rhTSH as effective and better tolerated in low-risk patients.	[9]
RCT	low-risk DTC; compared 30 mCi vs. 100 mCi with either THW or rhTSH	Ablation success rates: 87.1% with rhTSH vs. 86.7% with THW. For low-dose RAI (30 mCi) + rhTSH vs. high-dose RAI (100 mCi) + THW: 84.3% vs. 87.6%. Adverse events: 23% (rhTSH) vs. 30% (THW), *p* = 0.11. Time in hospital ≥ 3 days: 13% (rhTSH) vs. 36% (THW), *p* < 0.001.	[8]
RCT (ESTIMABL Trial)	Low-risk DTC; 752 patients randomized to 30 mCi vs. 100 mCi, with either rhTSH or THW	Complete ablation in 92% of evaluable patients overall. Ablation rates were similar between rhTSH (91.7%) and THW (92.9%). QoL is significantly better with rhTSH; hypothyroid symptoms are more common with THW. Lacrimal dysfunction occurred in 10% (rhTSH) vs. 22% (THW).	[10]
RCT	291 DTC patients undergoing remnant ablation with 30 mCi RAI after total thyroidectomy	Ablation success: 91.3% (rhTSH) vs. 91.0% (THW); *p* = 0.2061. Stimulated Tg after 12 months: 0.14 ± 0.05 ng/mL (rhTSH) vs. 0.18 ± 0.14 (THW); *p* = 0.1094. No difference in WBS outcomes. QoL significantly better with rhTSH: total score 4.2 ± 1.9 vs. 15.1–15.8 ± 3.1–4.1 in THW (*p* < 0.001).	[7]
Retrospective Cohort	647 DTC patients (mixed risk levels), Taiwan	“Excellent response” achieved in 80.5% (THW) vs. 76.5% (rhTSH); difference not statistically significant (*p* = 0.221). Subgroup analyses (age, sex, TNM stage, ETE, LN mets) also showed no significant differences. Concluded that rhTSH and THW offer comparable outcomes.	[15]
RCT (TRESON-01)	307 intermediate-risk DTC (T3/N1); all received 100 mCi RAI	RAI success rate: 43.8% (rhTSH) vs. 47.1% (THW); noninferior. Fewer adverse events with rhTSH: 30% vs. 59% (*p* < 0.001). Hypothyroid symptoms (e.g., weight gain, cold intolerance, constipation) were significantly lower with rhTSH.	[1]
Systematic Review and Meta-analysis	10 studies, 1929 patients with metastatic DTC	No difference in I-131 therapy outcomes between rhTSH (*n* = 953) and THW (*n* = 976). Initial response risk ratio: 1.02. Disease progression risk ratio: 0.97. Suggests that method of TSH stimulation does not impact therapeutic effectiveness in metastatic disease.	[2]
Retrospective Cohort	55 patients with distant metastatic DTC	No significant difference in PFS or OS between rhTSH and THW groups. Median PFS and OS not reported separately, but multivariate analysis showed that only age at diagnosis (*p* = 0.003) and total RAI activity (*p* = 0.03) were independently associated with progression and death.	[16]
Prospective Clinical Trial	366 DTC patients undergoing adjuvant RAI therapy	Whole-body half-life: 16.4 ± 4.6 h (rhTSH) vs. 19.3 ± 7.7 h (THW), *p* < 0.01. Thyroid uptake: 3.8% ± 1.6 (rhTSH) vs. 4.2% ± 1.8 (THW), *p* = 0.12. In patients with GFR < 60, the remaining-body dose was significantly lower with rhTSH (87 vs. 127 mGy, *p* < 0.01).	[24]
Systematic Review and Meta-analysis	5 studies; 953 thyroid cancer patients (321 rhTSH and 632 THW)	rhTSH reduced risk of long-term salivary gland dysfunction (LT-SGD). Pooled RR (fixed effect): 0.65 (95% CI: 0.49–0.86). Quality-adjusted model RR: 0.72 (95% CI: 0.54–0.96). Suggests small but significant protective effect, especially at higher RAI doses.	[26]
Multicenter Retrospective Cohort	404 DTC patients with nodal metastases (pT1–T3, N1, M0)	Disease-free status at 6–18 months: 75.1% (rhTSH) vs. 71.9% (THW); met noninferiority margin (<15%). At final follow-up (~30–37 months), complete response: 83.5% (rhTSH) vs. 81.5% (THW). No significant differences across subgroups (age, stage, LN burden).	[12]
Meta-analysis of 7 RCTs	1535 DTC patients post-thyroidectomy	Pooled ablation success: RR = 0.97 (95% CI: 0.94–1.01, *p* = 0.10); no significant difference between rhTSH and THW. Subgroup analysis showed no differences for low-dose or high-dose RAI. Suggests equivalent efficacy in remnant ablation.	[13]

QoL = quality of life; OS = overall survival; PFS = progression-free survival; Tg = thyroglobulin; RAI = radioactive iodine; WBS = whole-body scan; RR = risk ratio; NNT = number needed to treat; THW = thyroid hormone withdrawal; rhTSH = recombinant human thyroid-stimulating hormone; NS = not significant; ETE = extrathyroidal extension; LN mets = lymph node metastases; GFR = glomerular filtration rate.

**Table 2:** Summary comparison of rhTSH and thyroid hormone withdrawal (THW) for RAI preparation in differentiated thyroid cancer.

Parameter	rhTSH	THW	Reference
RAI Remnant Ablation Success	Equivalent to THW across risk levels and RAI doses	Equivalent	[7,8,9,10,11,12,14,15]
PFS	Comparable across risk levels	Comparable	[7,8,9,10,11,14]
Adverse Effects	Mild (injection site pain and rare nausea)	Common hypothyroid symptoms (fatigue, depression, cognitive slowing)	[1,8]
QoL	Preserved (euthyroid throughout)	Transient but significant decline during hypothyroid phase	[3,7,9,10]
Radiation to Non-Target Organs	Lower whole-body, marrow, kidney, and salivary exposure due to faster RAI clearance	Higher radiation exposure due to reduced renal clearance in hypothyroidism	[14,24,25]
Renal Function Impact	Preserved GFR	Transient GFR reduction, especially in elderly or CKD patients	[24]
Time to RAI Treatment	Short (2–3 days of preparation)	Long (3–4 weeks of hormone withdrawal required)	[3]
Convenience	High (outpatient injections and maintain routine)	Low (multiple visits, significant symptoms, time off work may be needed)	[2,3,11,23,27]
Cost and Access	Higher drug cost; may be limited in some healthcare systems	Low cost; no drug required	[3]

QoL = quality of life; PFS = progression-free survival; RAI = radioactive iodine; THW = thyroid hormone withdrawal; rhTSH = recombinant human thyroid-stimulating hormone; GFR = glomerular filtration rate.

## Abbreviations

The following abbreviations are used in this manuscript:

RAI	Radioactive Iodine
DTC	Differentiated Thyroid Cancer
TSH	Thyroid-Stimulating Hormone
THW	Thyroid Hormone Withdrawal
rhTSH	Recombinant Human Thyroid-Stimulating Hormone
QoL	Quality of Life
NIS	Sodium–Iodide Symporter
T3	Triiodothyronine
T4	Thyroxine
RCT(s)	Randomized Controlled Trial(s)
GFR	Glomerular Filtration Rate
PFS	Progression-Free Survival
OS	Overall Survival
Gy	Gray (unit of radiation dose)
FDA	Food and Drug Administration
ATA	American Thyroid Association
